# Correction: Martínez-Valverde et al. Health Needs Assessment: Chronic Kidney Disease Secondary to Type 2 Diabetes Mellitus in a Population without Social Security, Mexico 2016–2032. *Int. J. Environ. Res. Public Health* 2022, *19*, 9010

**DOI:** 10.3390/ijerph20054367

**Published:** 2023-02-28

**Authors:** Silvia Martínez-Valverde, Rodrigo Zepeda-Tello, Angélica Castro-Ríos, Filiberto Toledano-Toledano, Hortensia Reyes-Morales, Adrián Rodríguez-Matías, Juan Luis Gerardo Durán-Arenas

**Affiliations:** 1 Centro de Estudios Económicos y Sociales en Salud, Hospital Infantil de México Federico Gómez, Instituto Nacional de Salud, Mexico City 06720, Mexico; 2 Dirección de Prestaciones Económicas y Sociales, Instituto Mexicano del Seguro Social, Mexico City 06600, Mexico; 3 Unidad de Investigación en Epidemiología Clínica, Hospital de Pediatría, Centro Médico Nacional SXXI, Instituto Mexicano del Seguro Social, Mexico City 06720, Mexico; 4 Unidad de Investigación en Medicina Basada en Evidencias, Hospital Infantil de México Federico Gómez, Instituto Nacional de Salud, Mexico City 06720, Mexico; 5 Centro de Investigación en Sistemas de Salud, Instituto Nacional de Salud Pública, Cuernavaca 62100, Mexico; 6 Servicio de Nefrología, Hospital Angeles Metropolitano, Mexico City 06760, Mexico; 7 Departamento de Salud Pública, Facultad de Medicina, Universidad Autónoma de México, Mexico City 04510, Mexico

## Error in Table

In the original publication [1], there was a mistake in Table 3 as published. Table 3 has five sections, the fourth is “Progression to CKD” and the fifth is “Distribution”. 1. Please modify the text of the fourth section, instead of “Progression to CKD” change to “Progression to CKD (cases)”. 2. In the last “Distribution” section, the last column was duplicated. It should be deleted (the one that starts at 62.7%). 3. Then, all columns of the “Distribution” section should be shifted one place to the right to coincide with the year titles “2025 to 2032”. 4. Unify format, the lines of the two upper sections do not have horizonal lines, while the two lower sections do”. The corrected Table 3 appears below. The authors state that the scientific conclusions are unaffected. This correction was approved by the Academic Editor. The original publication has also been updated.

## Figures and Tables

**Table 3:** Health needs assessment of CKD secondary to T2 DM in patients without social security in Mexico (20–79 years of age).

Health Needs	^a/^2016	^b/^2017	^b/^2018	^b/^2019	^b/^2020	^b/^2021	^b/^2022	^b/^2023	^b/^2024
Diabetes population without social security	5,449,204	5,703,343	5,963,455	6,229,519	6,501,512	6,779,409	7,063,191	7,352,844	7,648,353
**Cases of chronic kidney disease secondary to T2 DM**									
Normoalbuminuria	4,026,612	4,124,684	4,225,015	4,327,494	4,432,015	4,538,472	4,646,773	4,756,831	4,868,561
Microalbuminuria	578,720	626,333	672,847	718,388	763,074	807,011	850,296	893,015	935,250
Macroalbuminuria	118,084	133,416	149,127	165,159	181,460	197,987	214,704	231,581	248,592
End-stage renal	28,750	31,562	34,415	37,307	40,236	43,199	46,194	49,216	52,264
Deaths associated with CV risk.	697,039	787,347	882,051	981,171	1,084,727	1,192,739	1,305,225	1,422,201	1,543,686
Distribution									
Normoalbuminuria	74%	72.3%	70.8%	69.5%	68.2%	66.9%	65.8%	64.7%	63.7%
Microalbuminuria	10.6%	11.0%	11.3%	11.5%	11.7%	11.9%	12.0%	12.1%	12.2%
Macroalbuminuria	2.2%	2.3%	2.5%	2.7%	2.8%	2.9%	3.0%	3.1%	3.3%
End-stage renal	0.53%	0.55%	0.58%	0.60%	0.62%	0.64%	0.65%	0.67%	0.68%
Deaths	12.8%	13.8%	14.8%	15.8%	16.7%	17.6%	18.5%	19.3%	20.2%
**Progression to CKD (cases)**	**^b/^2025**	**^b/^** **2026**	**^b/^** **2027**	**^b/^** **2028**	**^b/^** **2029**	**^b/^** **2030**	**^b/^ 2031**	**^b/^** **2032**	
Cohort of DM T2	7,949,693	8,256,841	8,569,754	8,888,350	9,212,529	9,542,175	9,877,143	10,217,299	
Normoalbuminuria	4,981,876	5,096,690	5,212,900	5,330,372	5,448,955	5,568,490	5,688,796	5,809,707	
Microalbuminuria	977,072	1,018,546	1,059,731	1,100,681	1,141,441	1,182,050	1,222,543	1,262,945	
Macroalbuminuria	265,716	282,938	300,243	317,622	335,065	352,566	370,121	387,724	
End-stage renal disease	55,335	58,425	61,533	64,657	67,795	70,945	74,108	77,280	
Deaths associated with CV risk	1,669,695	1,800,243	1,935,346	2,075,018	2,219,273	2,368,122	2,521,576	2,679,643	
Distribution									
Normoalbuminuria	62.7%	61.7%	60.8%	60.0%	59.1%	58.4%	57.6%	56.9%	
Microalbuminuria	12.29%	12.34%	12.37%	12.38%	12.39%	12.39%	12.38%	12.40%	
Macroalbuminuria	3.34%	3.43%	3.50%	3.57%	3.64%	3.69%	3.75%	3.79%	
End-stage renal disease	0.70%	0.71%	0.72%	0.73%	0.74%	0.74%	0.75%	0.76%	
Deaths	21.00%	21.80%	22.58%	23.35%	24.09%	24.82%	25.53%	26.23%	

Source: Authors’ estimates. ^a/^ Health Needs Assessment = T2 DM cases in 2016 that progressed to CKD; ^b/^ T2 DM cases (2017…2032) = Prevalence of T2 DM_(2016)_ + $\sum_{k\leq i}\mathrm{Incidence}\;\mathrm{of}\;T2\;\mathrm{DM}\;\mathrm{for}\;\mathrm{year}\;i$.
